# Supramolecular Nanofibers from Collagen-Mimetic Peptides Bearing Various Aromatic Groups at N-Termini via Hierarchical Self-Assembly

**DOI:** 10.3390/ijms22094533

**Published:** 2021-04-26

**Authors:** Tomoyuki Koga, Shinya Kingetsu, Nobuyuki Higashi

**Affiliations:** Department of Molecular Chemistry and Biochemistry, Faculty of Science and Engineering, Doshisha University, Kyotanabe, Kyoto 610-0321, Japan; ctwf0743@mail4.doshisha.ac.jp

**Keywords:** self-assembly, collagen-mimetic peptides, nanofibers, aromatic groups, small molecule binding, triple helix

## Abstract

Self-assembly of artificial peptides has been widely studied for constructing nanostructured materials, with numerous potential applications in the nanobiotechnology field. Herein, we report the synthesis and hierarchical self-assembly of collagen-mimetic peptides (CMPs) bearing various aromatic groups at the *N*-termini, including 2-naphthyl, 1-naphtyl, anthracenyl, and pyrenyl groups, into nanofibers. The CMPs (*R*-(GPO)*_n_*: *n* > 4) formed a triple helix structure in water at 4 °C, as confirmed via CD analyses, and their conformations were more stable with increasing hydrophobicity of the terminal aromatic group and peptide chain length. The resulting pre-organized triple helical CMPs showed diverse self-assembly into highly ordered nanofibers, reflecting their slight differences in hydrophobic/hydrophilic balance and configuration of aromatic templates. TEM analysis demonstrated that 2Np-CMP*_n_* (*n* = 6 and 7) and Py-CMP*_6_* provided well-developed natural collagen-like nanofibers and An-CMP*_n_* (*n* = 5–7) self-assembled into rod-like micelle fibers. On the other hand, 2Np-CMP*_5_* and 1Np-CMP*_6_* were unable to form nanofibers under the same conditions. Furthermore, the Py-CMP*_6_* nanofiber was found to encapsulate a guest hydrophobic molecule, Nile red, and exhibited unique emission behavior based on the specific nanostructure. In addition to the ability of CMPs to bind small molecules, their controlled self-assembly enables their versatile utilization in drug delivery and wavelength-conversion nanomaterials.

## 1. Introduction

Recently, significant efforts have been dedicated to the design of protein and peptide-based nanomaterials using a bottom-up approach with potential utility in biomedical and nanotechnology fields, such as tissue engineering, drug delivery, catalysis, and light harvesting [1,2,3,4,5,6]. Artificial oligopeptides are fascinating building units because of their (i) easy preparation by solid phase peptide synthesis (SPPS), (ii) structural and functional diversity by tuning the monomer sequence and chemical modification, (iii) biodegradability, and (iv) ability to hierarchically self-organize into well-defined nanostructures. It is well known that secondary structural motifs, including α-helices and β-sheets, can be designed intrinsically by the primary sequence of constituent amino acids. Therefore, a wide variety of nanoarchitectures such as nanofibers, nanotubes, vesicles, and nanospheres have been successfully fabricated by specifying the primary structure and controlling the interaction among secondary structural elements [1,2,3,4,5,6,7]. In particular, one-dimensional fibrous nanoassemblies are highly important not only as a new class of soft materials possessing a high specific surface area, light weight, and nanoporous structure, but also as a model for understanding biorelated self-assembly.

To date, various nanofibers have been developed by employing coiled-coil helices [8,9,10], β-sheets [11,12,13,14,15,16,17], and triple helix [18,19] peptides. Among such motifs, we focused on a collagen-like triple helix structure as a self-assembling unit. Collagen is the major protein constituent of diverse tissues, and its high natural abundance and good biocompatibility have accelerated the development of collagen as a biomaterial. Natural collagen is a rod-like macromolecule with a length of ca. 280 nm and a diameter of 1–2 nm, consisting of three parallel polyproline type II helices with unique repeats of X_aa_-Y_aa_-Gly (X_aa_ and Y_aa_ are Pro and Hyp in the most frequent triplet), and enables the formation of microscale-ordered fibrils [20,21,22]. In synthetic systems, triple-helix-based nanofibers have also been constructed from short CMPs by incorporating a “sticky-end” strategy [23,24] or specific assembly signals into both ends of a CMP triple helix, such as electrostatic interaction [25,26,27], π-π stacking [28,29], π-cation interaction [30,31] and metal-ligand coordination [32,33] to permit linear growth. More recently, our group demonstrated that photo-responsive azobenzene-terminated CMPs (9–24 mers) self-assembled into nanofibers and a hydrogel, in which the hydrophobic/hydrophilic balance had a significant effect on the fate of self-assembly [34] In this case, interestingly, a simple approach of introducing a hydrophobic group to only one end of the short CMP chain and adjusting the peptide length appropriately allowed the generation of well-developed nanofibers. Despite these successful advances, the structural factors required for such hierarchical self-assembly, as well as the mechanism, are not yet fully understood. Understanding the relationship between CMP structure and the consequent morphology and developing a novel self-assembling unit is challenging and valuable for the design of new functional nanobiomaterials.

In this study, we prepared short CMPs bearing various aromatic groups: 2-naphthyl (2Np), 1-naphthyl (1Np), 2-anthracenyl (An), and 1-pyrenyl (Py), which are capable of self-assembly into versatile nanofibers. Detailed analyses of the conformation and self-assembly behavior of the CMPs were conducted in water. Specifically, we investigated the effects of the structure and configuration of the terminal aromatic groups, peptide chain lengths, and external changes in temperature on the physicochemical behavior of the CMPs. Terminal modification of CMP with hydrophobic aromatic groups serves as a template for triple helix formation and a trigger to control nanoscale fibrous assembly, and is also useful for fabricating a wide variety of functional nanofibers. In fact, Py-terminated CMP nanofibers demonstrated the ability to encapsulate small hydrophobic molecules and acted as a thermo-responsive wavelength-conversion material.

## 2. Results and Discussion

### 2.1. Design and Synthesis of CMP Libraries

**Figure 1:**
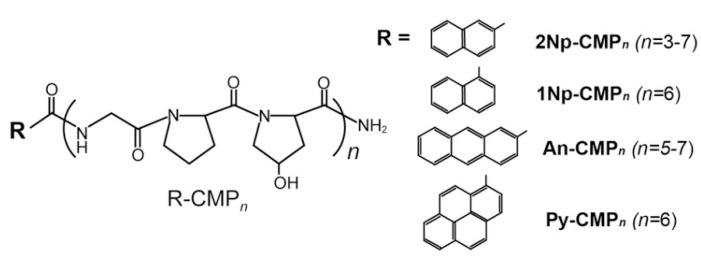
Chemical structures of the collagen-mimetic peptides bearing various aromatic groups at *N*-termini: 2Np-CMP*_n_* (*n* = 3–7), 1Np-CMP*_n_* (*n* = 6), An-CMP*_n_* (*n* = 5–7), Py-CMP*_n_* (*n* = 6).

All the CMPs employed in this study consist of repeated units of the Gly-Pro-Hyp triplet, (GPO)*_n_*, which is well-known to be the most stable sequence for the triple helix structure [22] and aromatic groups at the *N*-terminus. The *C*-termini were amidated to prevent ionization in water at neutral pH. We employed four types of aromatic molecules, 2Np, 1Np, An, and Py groups, each with a different hydrophobicity (Py > An > Np) and steric bulkiness. These aromatic moieties are expected to function as a template for tight alignment of the three strands via π-π stacking (i.e., triple helix formation) and as a trigger for further self-assembly [35]. In general, the stability of the collagen triple helix is strongly dependent on the peptide chain length, and short CMPs of *n* = 5 (15 mer) or shorter are unable to form a triple helix even with a GPO triplet (high triple helical propensity). However, the template-assisted folding strategy, namely the introduction of covalent or noncovalent links among the three peptide strands, has enabled the folding of short CMPs into a triple helix [36,37,38]. Based on these studies, ten distinct CMPs with systematically different chain lengths (*n* = 3–7) and aromatic groups were designed and synthesized (Figure 1). These CMP libraries possess a suitable hydrophobic/hydrophilic balance and terminal configuration for supramolecular assembly. All CMPs were successfully synthesized by standard SPPS using Fmoc-chemistry and identified by ^1^H-NMR and MALDI-TOF MS analysis (Figure S1).

### 2.2. Conformational and Self-Assembling Properties of 2Np-Terminated CMPs with Different Chain Lengths in Water

**Figure 2:**
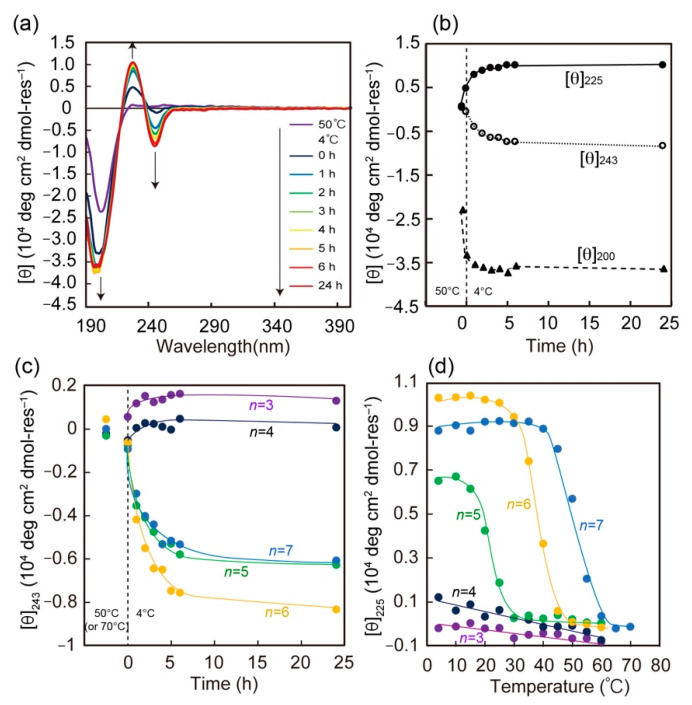
(**a**) CD spectral change of 2Np-CMP*_6_* in water (5% TFE) upon incubation at 4 °C after treatment at 50 °C (denatured form). (**b**) Time dependence of [θ]_200_, [θ]_225_ and [θ]_243_ values for aqueous 2Np-CMP*_6_* at 4 °C. (**c**) Time dependence of [θ]_243_ value for 2Np-CMP*_n_* (*n* = 3–7). (**d**) Temperature dependence of [θ]_225_ value of 2Np-CMP*_n_* (*n* = 3–7) in water (5% TFE). [2Np-CMP*_n_*] = 60 μM.

Initially, to assess the effect of peptide chain length on both secondary structure and thermal stability, circular dichroism (CD) studies of 2Np-CMP*_n_* (*n* = 3–7) were performed in water (Figure 2, Figures S2 and S3). Figure 2 shows the time dependence of the CD spectra for 2Np-CMP*_6_* in water (5% TFE) at 4 °C. At 50 °C, the spectrum initially shows a random coil pattern with a negative maximum at approximately 200 nm. When the sample solution was cooled to 4 °C, the conformation changed to a triple helix, as evident from the presence of a maximum at 225 nm and a minimum at 200 nm [37]. Meanwhile, an induced CD effect was clearly observed at 243 nm (negative) and 225 nm (positive) based on a twisted arrangement of 2Np moiety (λ_max_ = 235 nm), reflecting the spiral structure of the triple helix [39]. In our previous study [34], a similar induced CD effect was also observed for an azobenzene-terminated CMP system by triple helix formation when the azobenzene group was directly attached to the CMP chain end without a spacer. Figure 2b shows plots of molar ellipticity at 200 nm ([θ]_200_), 225 nm ([θ]_225_), and 243 nm ([θ]_243_) as a function of time. As time elapses, the [θ]_200_ value decreases rapidly and reaches a constant value within 2 h, whereas induced CD signals ([θ]_243_ and [θ]_225_) continue to change over 6 h. This time lag suggests that the triple helix foldamer behaves as a precursor to further supramolecular assembly triggered by specific interactions of *N*-terminal 2Np groups and/or *C*-terminal amide groups (morphological study; described later). Notably, the conformational properties of 2Np-CMP*_n_* are chain-length-dependent; 2Np-CMP*_n_* with shorter lengths of *n*=3 or 4 cannot form a triple helix assembly even after incubation for 24 h at 4 °C (Figure 2c and Figure S2). To elucidate the triple helix stability of 2Np-CMP*_n_*, their melting temperatures (*T*_m_) were evaluated by monitoring the change in [θ]_225_ values as a function of temperature (Figure 2d). The cooperative melting curves were obtained only in the case of longer 2Np-CMP*_n_* (*n* = 5–7), and the *T*_m_ values gradually decreased by approximately 10–15 °C upon decreasing the repeat number (*n*) of the GPO triplet from *n* = 7 to 5.

**Figure 3:**
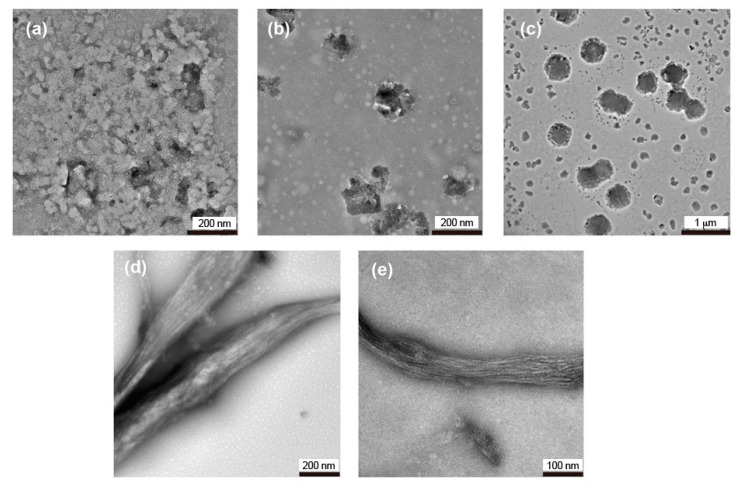
TEM images of 2Np-CMP*_n_* (*n* = 3–7) assemblies formed in water (5% TFE) at 4 °C (*n* = 3 (**a**), *n* = 4 (**b**), *n* = 5 (**c**), *n* = 6 (**d**), *n* = 7 (**e**)). [2Np-CMP*_n_*] = 60 μM. The images were obtained by staining with 1% phosphotungstic acid.

To comprehensively understand the self-assembly of 2Np-CMPs in water, morphological observations of the samples after incubation at 4 °C were conducted by TEM analysis (Figure 3). Remarkably, 2Np-CMP*_n_* (*n* = 6 and 7) self-assembled into fiber structures with lengths on the order of micrometers (Figure 3d,e). These fibrils are bundles of several protofibrils with a diameter of approximately 4 nm and are arranged laterally, similar to natural collagen fibers [21,40,41]. It seems that five triple helix precursors are aligned one-dimensionally because of hydrophobic interaction between the *N*-terminal 2Np groups and hydrogen bonding between the *C*-terminal amide groups (see Section 2.3). Hydrophobic defects derived from aromatic clusters in the nanofiber likely cause bundle formation to stabilize the resultant assemblies. In contrast, 2Np-CMP*_3_* does not form any specific assembly (Figure 3a), and 2Np-CMP*_n_* (*n* = 4 and 5) shows the formation of amorphous and/or sheet-like [42] aggregates (Figure 3b,c). Note that a sheet-like assembly with a height of 3.5–4.5 nm was partially observed in the AFM analysis of 2Np-CMP*_n_* (*n* = 5) (Figure S4). Thus, even a slight difference in hydrophobic/hydrophilic balance has a significant influence on the self-assembly behavior of the aqueous 2Np-CMP molecular system.

### 2.3. Effect of Terminal Aromatic Groups on Conformation and Self-Assembly

**Figure 4:**
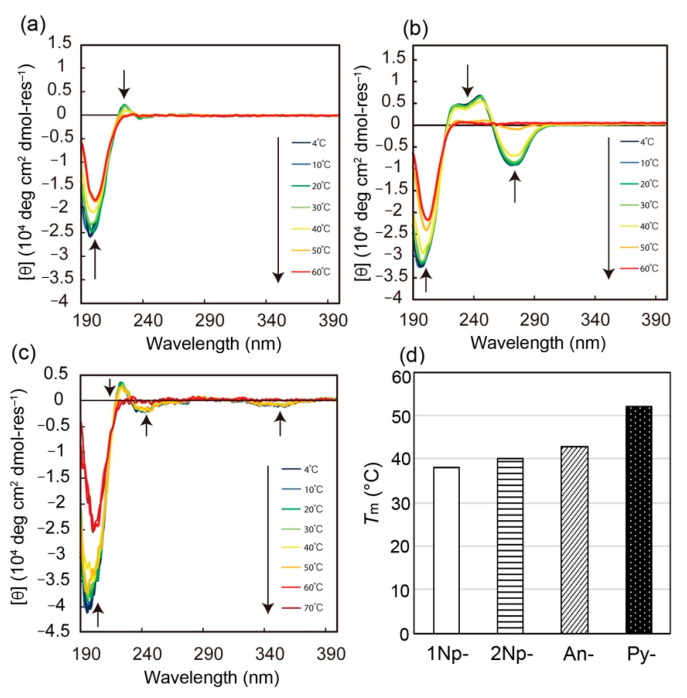
CD spectral changes of 1-Np-CMP*_6_* (**a**), An-CMP*_6_* (**b**) and Py-CMP*_6_* (**c**) in water (5% TFE) upon heating. [CMPs] = 60 μM. (**d**) Comparison of *T*_m_ for various aromatic group-terminated CMPs (*n* = 6) evaluated from CD analyses.

**Figure 5:**
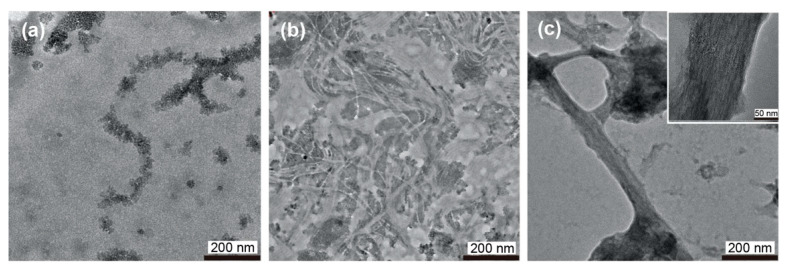
TEM images of 1Np-CMP*_6_* (**a**), An-CMP*_6_* (**b**) and Py-CMP*_6_* (**c**) assemblies formed in water (5% TFE) at 4 °C. [CMPs] = 60 μM. The images were obtained by staining with 1% phosphotungstic acid.

**Figure 6:**
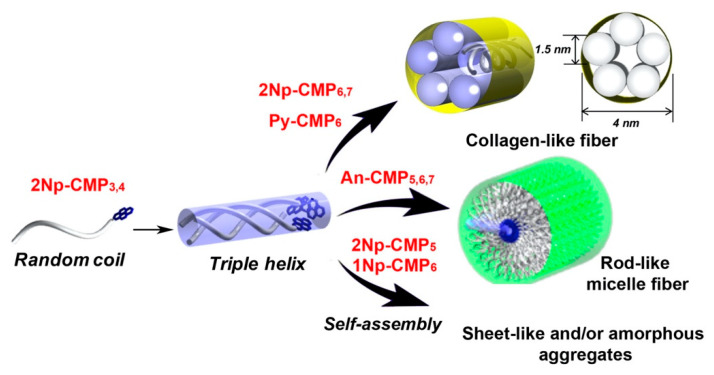
Schematic illustration of a hierarchical self-assembly model for a series of CMPs bearing various aromatic groups.

We next investigated the impact of terminal aromatic groups on the conformation and self-assembly of the CMPs. Figure 4 shows the conformational properties of 1Np-CMP*_n_*, An-CMP*_n_*, and Py-CMP*_n_* with the same chain length of *n* = 6 (18 mer) in water (5% TFE). In all cases, conformational transitions from triple helix to random coil are observed upon heating, as evident from the decrease and increase in [θ]_225_ and [θ]_200_ values, respectively. Similar to the behavior of the 2Np-CMP*_n_*, obvious induced CD signals based on triple helical conformation—except 1Np-CMP*_6_*, which does not have strong absorption in the 220–240 nm region—are also observed in the absorption regions of the corresponding aromatic groups (λ_max_ = 260 nm (An) and λ_max_ = 245 nm and 342 nm (Py)). Interestingly, enhanced π-π interaction leads to an increase in the stability of the triple helical conformation, and the *T*_m_ of Py-CMP*_6_* reaches 52 °C (Figure 4d). In addition, there is a difference in *T*_m_ between 2Np- and 1Np-CMPs, demonstrating the importance of the configuration of the aromatic template. Indeed, depending on the character of the terminal aromatic groups, the CMPs show diverse self-assembly behavior (Figure 5). Unlike the case of 2Np-CMP*_6_*, fiber assembly is not observed for 1Np-CMP*_6_*. In contrast, Py-CMP*_6_* self-assembles into collagen-like nanofibers composed of bundles of 4 nm protofibrils, similar to 2Np-CMP*_6_*. Notably, the size and shape of the An-CMP*_6_* nanofibers are quite different from those of 2Np-CMP*_6_* and Py-CMP*_6_*; that is, nanofibers with a homogeneous diameter of 12 nm are observed (Figure 5b). Given that the theoretical An-CMP*_6_* length, assuming a complete triple helical conformation, is approximately 5.7 nm, the observed diameter of the nanofiber corresponds is about twice the molecular length. Thus, it can be concluded that An-CMP*_6_* self-assembles into rod-like micelle fibers with hydrophobic aromatic cores and shells of hydrophilic peptide segments via triple helix formation. The diameters of the An-CMP*_n_* nanofibers were found to increase linearly with increasing chain length (10 nm (15 mer), 12 nm (18 mer), and 14 nm (21 mer)) (Figure S5). Overall, diverse self-assembly was successfully achieved by manipulating the hydrophobic/hydrophilic balance and the aromatic template (Figure 6).

### 2.4. Encapsulation of Nile Red into Py-CMP Nanofiber for Thermo-Responsive Drug Delivery Systems (DDSs) and Wavelength-Conversion Materials

**Figure 7:**
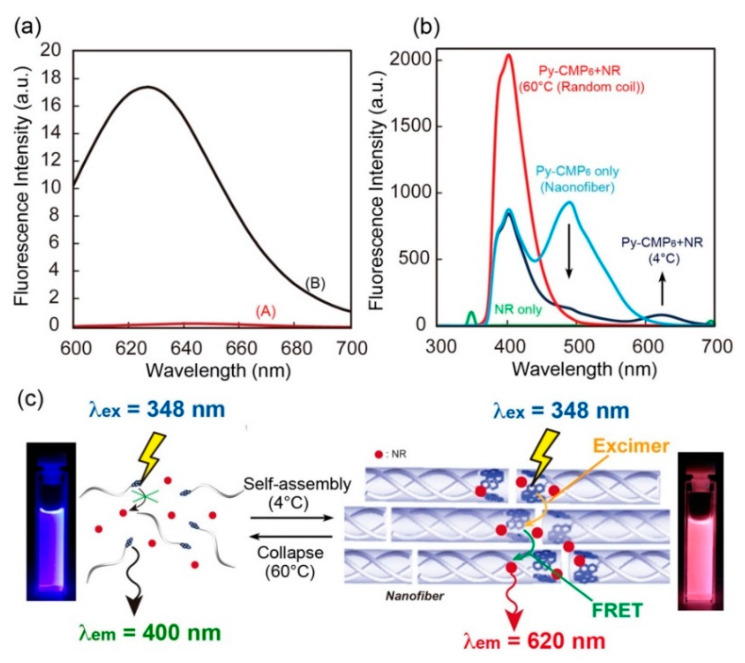
(**a**) Fluorescence spectra of NR (2 μM) in the absence (A) and presence (B) of Py-CMP*_6_* nanofiber in water (1% EtOH, 5% TFE) at 4 °C. λ_ex_ = 580 nm. [Py-CMP*_6_*] = 60 μM. (**b**) Fluorescence spectra of NR/Py-CMP*_6_* nanofiber in water at 4 °C and 60 °C. λ_ex_ = 348 nm. The spectra of aqueous NR and Py-CMP*_6_* nanofiber (4 °C) are also included for comparison. (**c**) Schematic model for the conversion of energy from shorter to longer wavelengths in Py-CMP*_6_* nanofiber. Photographs show NR/Py-CMP*_6_* nanofiber aqueous solutions at 4 °C (right) and 60 °C (left) under UV lamp (365 nm).

Because these CMP-based nanofibers possess hydrophobic domains capable of binding small molecules, we assessed their ability to encapsulate Nile red (NR) as a hydrophobic and fluorescent model compound [43,44,45]. Figure 7a shows the fluorescence emission spectra of NR (2 μM) in the absence and presence of Py-CMP*_6_* nanofibers (60 μM) in water (1% EtOH, 5% TFE, 4 °C) when excited at 580 nm. The fluorescence of NR is known to be strongly induced by the polarity of the surrounding environment, and the λ_em_ shifts toward a shorter wavelength with an increasingly hydrophobic environment [46]. A slight emission of free NR is observed in water at 645 nm. In the presence of Py-CMP*_6_* nanofibers, the fluorescence signal increases significantly and is blue-shifted to λ_em_ = 630 nm, suggesting that NR is present in the hydrophobic domains of the nanofiber. Thus, the introduction of the Py group at the CMP terminus enhances not only the overall thermostability of the fiber but also the binding of small hydrophobic molecules.

Finally, we investigated the fluorescence properties of the Py-CMP*_6_*/NR mixed molecular system based on its unique self-assembled structure. As described above, Py-CMP*_6_* assembles into collagen-like nanofiber bundles in aqueous media at 4 °C. During self-assembly, emission switching occurs from the monomer (λ_em_ = 402 nm) to excimer (λ_em_ = 490 nm) due to π-stacking of Py groups (λ_ex_ = 348 nm) (Figure S6). Figure 7b shows the fluorescence emission spectra of Py-CMP*_6_*/NR at 4 °C (nanofiber form) and 60 °C (random coil form) when excited at 348 nm. Fluorescence emission from NR is clearly observed at 620 nm in the Py-CMP*_6_*/NR nanofiber system (4 °C), demonstrating that the electron energy of the Py group excited at 348 nm is transferred to NR via Py excimer formation (Figure 7c). As a control, we confirmed that no fluorescence at 620 nm is observed in solutions with only NR or Py-CMP*_6_* nanofibers when excited at 348 nm. More importantly, such conversion of energy from shorter to longer wavelengths can be achieved only in the triple-helix-based nanofiber state and exhibits completely different behavior at 60 °C; that is, it disappears with the collapse of the nanofibers, resulting in a color change. This feature would be useful in designing novel thermo-responsive optical nanomaterials and artificial extracellular matrix (ECM) materials with damage detecting ability.

## 3. Experimental Section

### 3.1. Materials

*N*,*N*-Dimethylformamide (DMF), methanol, ethanol (EtOH), diethylether, acetone, sodium hydroxide (NaOH), D_2_O, 2,2,2-trifluoroethanol (TFE) were purchased from Nacalai Tesque. Dichloromethane, 1-naphthoic acid (1-Np), 2-naphthoic acid (2-Np), *N,N’*-diisopropylcarbodiimide (DIPC) and Nile Red (NR) were purchased from Wako Pure Chemical (Osaka, Japan). Fmoc-Hyp(tBu), Fmoc-Gly, Fmoc-NH-SAL MBHA Resin (0.67 mmol/g), trifluoroacetic acid (TFA) and 1-hydroxy-7-azabenzotriazole (HOAt) were purchased from Watanabe Chemical Industries (Hiroshima, Japan). Fmoc-Pro was purchased from Peptide Institute. 2-Anthracenecarboxylic acid (An) and 1-pyrenecarboxylic acid (Py) were purchased from Tokyo Chemical Industries (Tokyo, Japan). 2,5-Dihydroxybenzoic acid (DHBA) were purchased from Sigma Aldrich (St Louis, MO, USA). All reagents were used as received.

### 3.2. Measurements

^1^H-NMR spectra were acquired using a JEOL JNM-ECA-500 (JEOL Ltd., Tokyo, Japan) spectrometer (500 MHz). Matrix-assisted laser desorption ionization-time-of-flight MS (MALDI-TOF MS) analyses were carried out on an Autoflex Speed instrument (Bluker Daltonics, Billerica, MA, USA) using DHBA as a matrix. CD spectra were recorded on a J-820 spectropolarimeter (JASCO Ltd., Tokyo, Japan) equipped with a Peltier-type thermostatic cell holder coupled with a PTC-423L controller under a nitrogen atmosphere. Experiments were performed in a quartz cell with a path length of 1 mm. All CMPs were dissolved in TFE as a stock solution before the conformation and self-assembly assays. Sample solutions of the CMPs (final concentration: 60 μM, TFE content: 5 vol%) were prepared by diluting the stock solution with pure water. The temperature dependence of the CD spectra was measured over the range 190–400 nm at 4 °C to 70 °C (1 °C/min). UV-vis spectra were recorded on a V-650 spectrometer (JASCO Ltd., Tokyo, Japan). Fluorescence spectra were recorded in a quartz cell with a 1 cm path length using an FP-8300 spectrofluorometer (JASCO Ltd., Tokyo, Japan) equipped with a Peltier-type thermostatic cell holder coupled with an ETC-815 controller. The experiments were conducted in the wavelength range 300–700 nm at 4 °C to 60 °C (1 °C/min) (λ_ex_ = 580 nm for NR-encapsulation study and λ_ex_ = 348 nm for energy conversion experiments). Samples were prepared using the following method: NR was dissolved in ethanol. NR solution was added to 5 mL of a Py-CMP_6_ solution (final NR concentration: 2 μM, CMP concentration: 60 μM, water:TFE:EtOH = 94:5:1 by vol.) and incubated at 4 °C for 24 h prior to the experiment. TEM images were collected on a JEOL JEM2100F (JEOL Ltd., Tokyo, Japan) instrument at an accelerating voltage of 200 kV. After a small volume of CMP aqueous solution was applied to a carbon-coated copper TEM grid for 20 min at 4 °C, excess solution was blotted using a filter paper and the sample was stained with a phosphotungstic acid aqueous solution (1 wt%). The samples were then dried in a covered container. AFM image was collected on a SPM9700 (Shimazu Co., Kyoto, Japan) operated by tapping using a silicon tip (MPP-11100). An aliquot of the aqueous solution of CMP assembly was placed on freshly cleaved mica at 4 °C. After adsorption for 20 min, the excess solution was removed by absorption with filter paper.

### 3.3. Synthesis of Collagen-Mimetic Peptides Bearing Various Aromatic Groups at N-Termini

Collagen-mimetic peptides used as self-assembling units bearing the aromatic groups 1Np, 2Np, An, and Py at the *N*-termini were prepared by solid-phase peptide synthesis using 9-fluorenylmethoxycarbonyl (Fmoc) chemistry. The target sequence was prepared on an Fmoc-NH-SAL MBHA resin using Fmoc-Hyp(tBu), Fmoc-Pro, and Fmoc-Gly (3 equiv.) in DMF. 1-Hydroxy-7-azabenzotriazole (HOAt) (3 equiv.) and 1,3-diisopropylcarbodiimide (DIPC) (3 equiv.) were used for coupling, and piperidine (20 vol%) in DMF was used for Fmoc removal. The resultant resin was treated with various aromatic molecules: 1-naphthoic acid, 2-naphthoic acid, 2-anthracenecarboxylic acid, and 1-pyrenecarboxylic acid (3 equiv.), HOAt (3 equiv.) and DIPC (3 equiv.) in DMF. After the modification, the target peptide was obtained by cleaving it from the resin using TFA/DCM (9:1 in vol.). The obtained peptides were identified by ^1^H NMR and MALDI-TOF MS analysis (Figure S1).

**2Np-CMP*_n_***; Yield: 88% (*n* = 3), 52% (*n* = 4), 92% (*n* = 5), 75% (*n* = 6), 95% (*n* = 7), MALDI-TOF MS: 995.0 [M+Na]^+^/996.0 [M+Na]^+^_calcd._ (*n* = 3), 1262.0 [M+Na]^+^/1263.3 [M+Na]^+^_calcd._ (*n* = 4), 1531.8 [M+Na]^+^/1530.6 [M+Na]^+^_calcd._ (*n* = 5), 1798.5 [M+Na]^+^/1797.9 [M+Na]^+^_calcd._ (*n* = 6), 2065.9 [M+Na]^+^/2065.2 [M+Na]^+^_calcd._ (*n* = 7). ^1^H NMR (D_2_O, DSS): 1.9–2.6 ppm (Pro-β, Pro-γ, Hyp-β), 3.2–4.2 ppm (Pro-δ, Hyp-δ, Gly-α), 4.4–4.8 ppm (Hyp-γ, Hyp-α, Pro-α, overlapped with D_2_O), 7.0–8.5 ppm (aromatic ring of naphthalene).

**1Np-CMP*_n_* (*n* = 6)**; Yield: 95%, MALDI-TOF MS 1798.4 [M+Na]^+^/1797.9 [M+Na]^+^_calcd._

^1^H NMR (D_2_O, DSS): 1.9–2.6 ppm (Pro-β, Pro-γ, Hyp-β), 3.2–4.2 ppm (Pro-δ, Hyp-δ, Gly-α), 4.4–4.8 ppm (Hyp-γ, Hyp-α, Pro-α, overlapped with D_2_O), 7.0–8.3 ppm (aromatic ring of naphthalene).

**An-CMP*_n_***; Yield: 92% (*n* = 5), 70% (*n* = 6), 93% (*n* = 7), MALDI-TOF MS 1580.8 [M+Na]^+^/1580.7 [M+Na]^+^_calcd._ (*n* = 5), 1849.5 [M+Na]^+^/1848.0 [M+Na]^+^_calcd._ (*n* = 6), 2116.9 [M+Na]^+^/2115.3 [M+Na]^+^_calcd._ (*n* = 7).

^1^H NMR (D_2_O, DSS) 1.9–2.6 ppm (Pro-β, Pro-γ, Hyp-β), 3.0–4.1 ppm (Pro-δ, Hyp-δ, Gly-α), 4.4–4.8 ppm (Hyp-γ, Hyp-α, Pro-α, overlapped with D_2_O), 7.2–8.6 ppm (aromatic ring of anthracene).

**Py-CMP*_n_* (*n* = 6)**; Yield: 94%, MALDI-TOF MS 1873.6 [M+Na]^+^/1872.0 [M+Na]^+^_calcd._^1^H NMR (D_2_O, DSS) 1.9–2.6 ppm (Pro-β, Pro-γ, Hyp-β), 3.0–4.1 ppm (Pro-δ, Hyp-δ, Gly-α), 4.2–4.8 ppm (Hyp-γ, Hyp-α, Pro-α, overlapped with D_2_O), 8.0 ppm (aromatic ring of pyrene (broad)).

## 4. Conclusions

In summary, we proposed a unique strategy for constructing CMP-based nanofiber architectures. Ten types of CMPs with different terminal aromatic groups and chain lengths were successfully synthesized using a simple SPPS. Our studies demonstrated that both the type of aromatic template and the length of the peptide segment can not only affect the conformational properties but also, more importantly, influence the supramolecular assembly, resulting in the generation of two highly ordered types of nanofibers with different shapes and sizes based on the packing of constituent triple helical amphiphiles (i.e., natural collagen-like fibers and rod-like micelle fibers). In addition, the Py-CMP*_6_* nanofiber was found to act efficiently as a nanocontainer for hydrophobic small molecules for DDS and wavelength-conversion materials [47]. We believe that these findings will provide important insights into the fine-tuning of peptide-based supramolecular nanofibers with potential applications in nanotechnology and biomedical fields.

## Supplementary Materials

The following are available online at https://www.mdpi.com/article/10.3390/ijms22094533/s1. The Supporting Information is available free of charge. Supplemental figures (Figures S1–S6) (PDF).
