# Supplementary material for: Supramolecular Nanofibers from Collagen-Mimetic Peptides Bearing Various Aromatic Groups at N-Termini via Hierarchical Self-Assembly
Source: Int J Mol Sci. 2021 Apr 26;22(9):4533. doi: 10.3390/ijms22094533 (PMC8123610; doi:10.3390/ijms22094533)
Supplement: Supplementary file 1 [file ijms-22-04533-s001.zip › ijms-1179998-supplementary.pdf]

[Supplemental Materials]

Supramolecular Nanofibers from Collagen-mimetic Peptides Bearing Various Aromatic Groups at N-Termini via Hierarchical Self-assembly

Tomoyuki Koga,* Shinya Kingetsu, Nobuyuki Higashi*

Department of Molecular Chemistry and Biochemistry, Faculty of Science and Engineering, Doshisha University, Kyotanabe, Kyoto 610-0321, JAPAN

Corresponding Author

*E-mail: tkoga@mail.doshisha.ac.jp (T. K.)

*E-mail: nhigashi@mail.doshisha.ac.jp (N. H.)

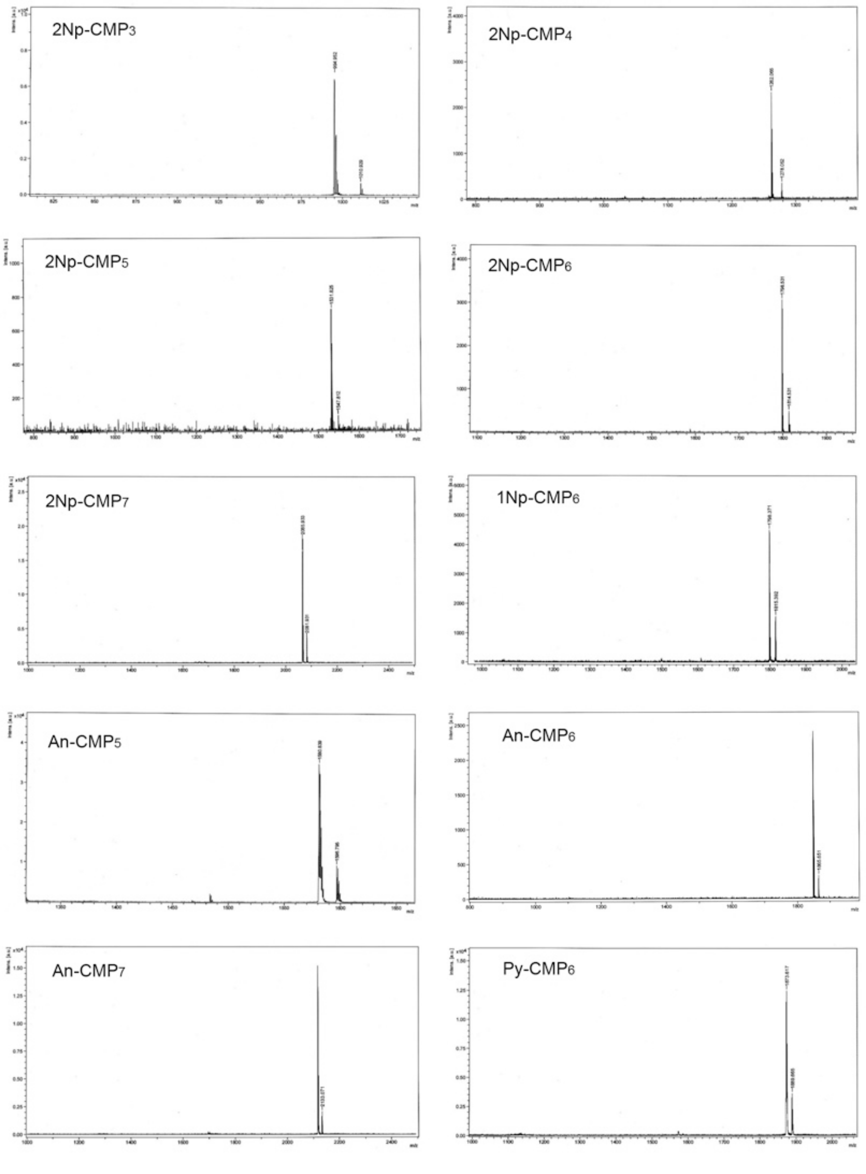

Figure S1. MALDI TOF MS spectra of 2Np-CMP$_n$ (n=3–7), 1Np-CMP$_6$, An-CMP$_n$ (n=5–7) and Py-CMP$_6$. Matrix: DHBA.

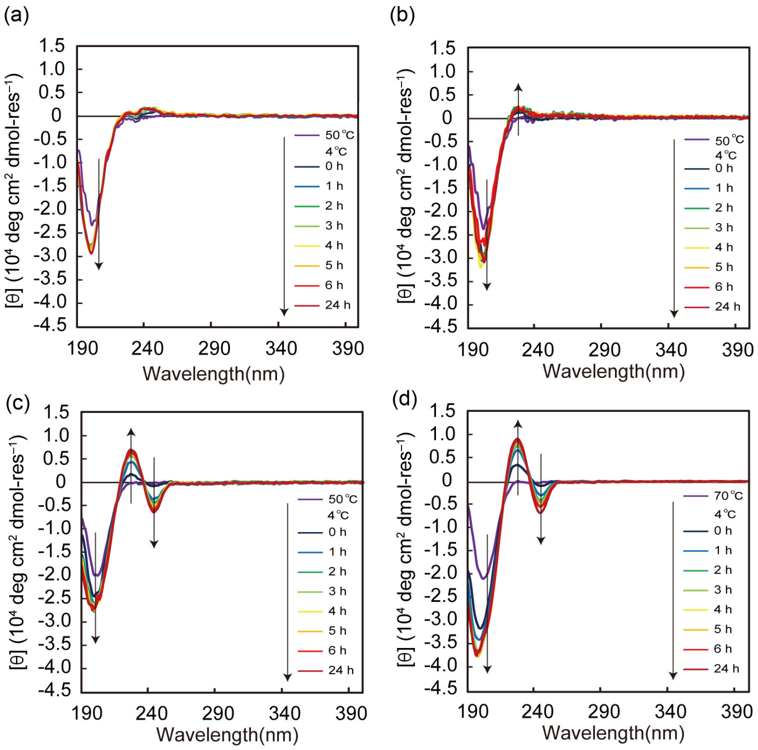

Figure S2. CD spectral change of 2Np-CMP$_n$ (n=3 (a), n=4 (b), n=5 (c), n=7 (d)) in water (5% TFE) upon incubation at 4 °C after treatment at 50 °C or 70 °C (denatured form). [2Np-CMP$_n$]=60 μM.

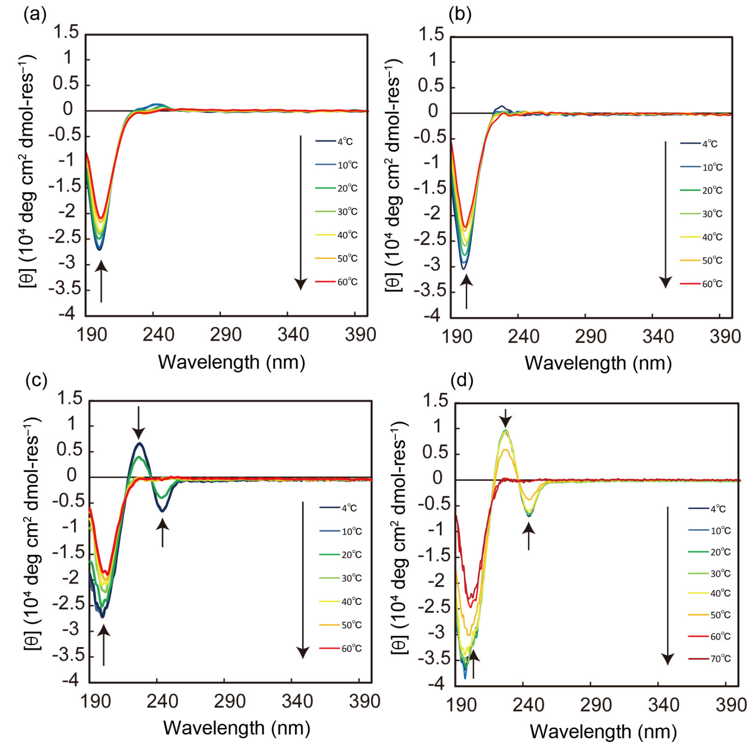

Figure S3. Temperature dependence of CD spectra of 2Np-CMP$_n$ (n=3 (a), n=4 (b), n=5 (c), n=7 (d)) in water (5% TFE). [2Np-CMP$_n$]=60 μM.

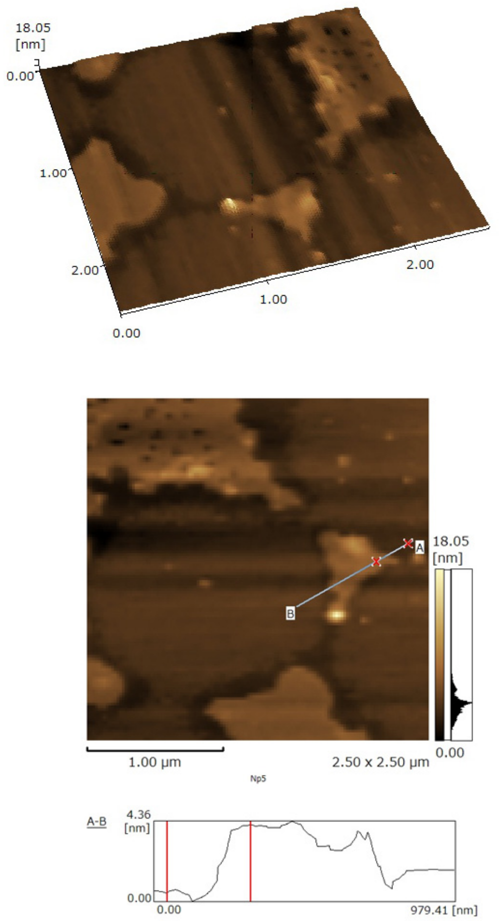

Figure S4. AFM image of 2Np-CMP$_n$ (n=5) assemblies formed in water (5% TFE) at 4 °C. [2Np-CMP$_n$]=60 µM.

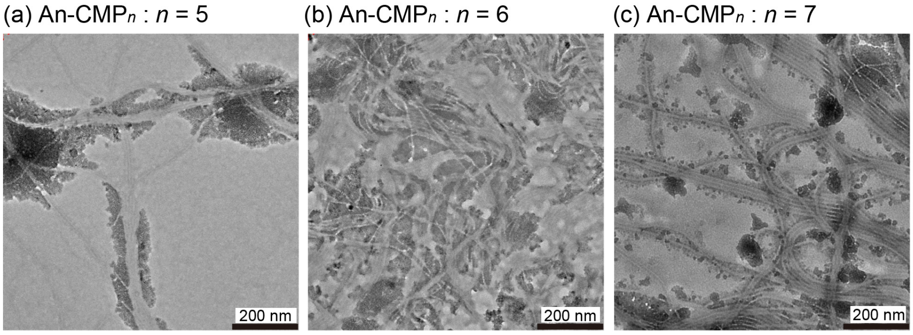

Figure S5. TEM images of An-CMP$_n$ (n=5 (a), n=6 (b), n=7 (c)) assemblies formed in water (5% TFE) at 4 °C. [An-CMP$_n$]=60 μM. The images were obtained by staining with 1% phosphotungstic acid.

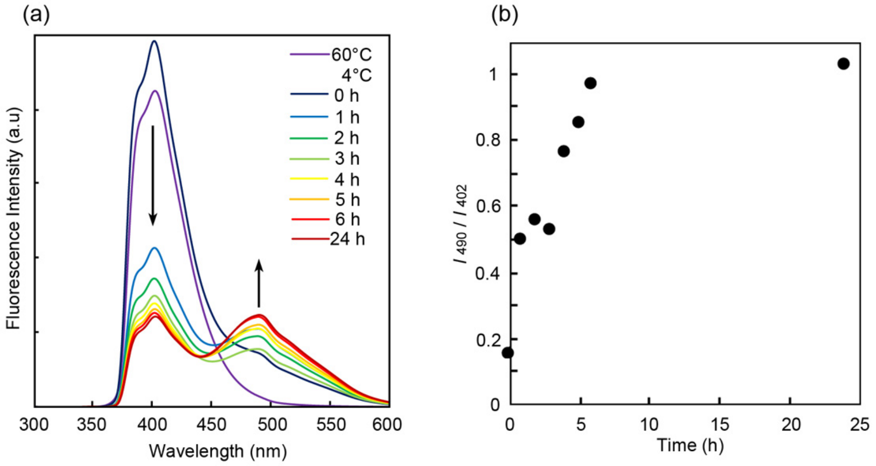

Figure S6. (a) Fluorescence spectral change of Py-CMP$_6$ in water (5% TFE) upon incubation at 4 °C after treatment at 60 °C. [Py-CMP$_6$]=60 μM. (b) Time-dependence of I_{490} (excimer)/ I_{402} (monomer) value. λ_{ex}=348 nm.
